# Palliative Approach to Care Education for Multidisciplinary Staff of Long-Term Care Homes: A Pretest Post-Test Study

**DOI:** 10.1177/23337214231158470

**Published:** 2023-02-22

**Authors:** Shirin Vellani, Vanessa Maradiaga Rivas, Maria Nicula, Stephanie Lucchese, Julia Kruizinga, Tamara Sussman, Sharon Kaasalainen

**Affiliations:** 1 Faculty of Health Sciences, School of Nursing, McMaster University, Hamilton, ON, Canada; 2 Faculty of Arts, School of Social Work, McGill University, Montreal, QC, Canada

**Keywords:** long-term care, advance care planning, palliative approach to care, educational workshop

## Abstract

This study used a single-group pre-test and post-test design to evaluate an educational workshop for multidisciplinary staff working in long-term care homes on implementing a palliative approach to care and perceptions about advanced care planning conversations. Two outcomes were measured to assess the preliminary efficacy of the educational workshop at baseline and 1-month post-intervention. Knowledge regarding implementing a palliative approach to care was assessed using the End-of-Life Professional Caregivers Survey and changes in staff perception toward ACP conversations were assessed using the Staff Perceptions Survey. Findings suggest that staff experienced an improvement in self-reported knowledge regarding a palliative approach to care (*p* ≤ .001); and perceptions of knowledge, attitude, and comfort related to advance care planning discussions (*p* ≤ .027). The results indicate that educational workshops can assist in improving multidisciplinary staff’s knowledge about a palliative approach to care and comfort in carrying out advance care planning discussions with residents, family care partners, and among long-term care staff.

**What this paper adds?**

- This study includes perspectives from multidisciplinary long-term care staff
- It presents details on an educational intervention from an existing program called Strengthening a palliative approach in long-term care (SPA-LTC)
- Educational interventions increase knowledge and perception about a palliative approach to care for multidisciplinary staff in long-term care which may positively impact their practice

**Applications of study findings:**

- Educational initiatives centered on improving knowledge and comfort with a palliative approach to care and advanced care planning for long-term care multidisciplinary staff should be made priority for onboarding new staff and maintaining their level of comfort and skillset
- Future evaluation of educational workshops in long-term care exploring frequency, mode and quality indicators for advanced care planning discussions.

## Introduction

Older adults are susceptible to frailty and the development of multiple chronic illnesses (e.g., dementia, kidney disease, cancer) (Burch et al., 2014). About 7% of those aged 65 and older and 32% of those aged 85 years and older reside in long-term care (LTC) homes and other assisted living facilities (Statistics Canada, 2012, 2017) to meet their daily activities needs due to cognitive and functional disabilities. Those residing in LTC homes receive round the clock personal and nursing care as they are unable to thrive in their own homes due to inadequate health and social care services (Nicolle, 2000). Older residents of LTC homes are often complex with about 90% having some form of cognitive impairment including dementia, and over 60% receive 10 or more prescription medications suggesting presence of multimorbidity (Ontario Long-Term Care Association, 2019); and most die within 2 years of admission (Vossius et al., 2022).

A palliative approach to care can promote a comprehensive person-centered care for residents and their care partners and assist with seamless transition to end-of-life care. A palliative approach to care provides for residents’ needs throughout their life trajectory in the LTC homes including advance care planning (ACP) discussions, management of distressing symptoms, psychosocial and spiritual care as well as grief and bereavement support for family, other residents and staff (Kaasalainen et al., 2019). There is higher evidence of an increased risk for decisional incapacity in older adults due to the increasing incidence of cognitive impairment, making it critical to promote early ACP discussions (Siu et al., 2020; Sussman, Pimienta, & Hayward, 2021). The integration of ACP discussions as a critical component of residents’ care plan has demonstrated positive outcomes for residents, family care partners and the healthcare system such as improved quality of life and death; satisfaction with the quality of care, and reduced unnecessary hospitalization (Siu et al., 2020; Sussman, Pimienta, & Hayward, 2021).

Advance care planning involves reflecting on one’s values and wishes for future care when one may be unable to express them (e.g., due to cognitive impairment or high levels of frailty) and sharing them with trusted individuals (e.g., family/friends) (Advance Care Planning Canada, 2022; Sudore et al., 2017). These conversations are relevant for all adults regardless of their age or stage of the disease (Sudore et al., 2017). ACP is a critical component to implementing a palliative approach to care in long-term care (LTC) homes; it allows residents and their care partners such as family members or friends to be better prepared for end-of-life (EOL) care and decision-making (Gilissen et al., 2018; Sudore et al., 2017; Vellani, Zuniga et al., 2022). However, many LTC residents are not offered opportunities to participate in ACP discussions prior to or upon their admission to LTC (Hunter et al., 2020; Sussman et al., 2020; Sussman, Kaasalainen et al., 2021). Many receive life-sustaining treatments including admission to critical care in the last 3 months of their life (Chaudhuri et al., 2017). One factor that is widely considered to contribute to the poor uptake of ACP in LTC is the lack of knowledge and sense of discomfort with these conversations involving residents and their family care partners, among all categories of LTC staff (Hunter et al., 2020; Jeong et al., 2011; Kaasalainen et al., 2017; Lin et al., 2019; Spacey et al., 2021).

Formal education regarding ACP can assist in promoting LTC staff engagement in such discussions with residents and their care partners (Beck et al., 2017; Gilissen et al., 2018; Huang et al., 2020; Iida et al., 2021; Noh et al., 2021). Yet, few studies of formal education regarding ACP have been evaluated for multidisciplinary LTC staff within Canada (Cloutier et al., 2021; Kaasalainen, Mccleary et al., 2021; Pereira et al., 2021; Sarakbi et al., 2022). The purpose of this study was to evaluate a multidisciplinary staff educational workshop about the implementation of a palliative approach to care with a focus on ACP within three LTC homes in two cities in Southern Ontario. The workshop was designed to increase the staff’s knowledge and enhance their skills regarding communication with residents, family care partners, and other staff about ACP through interactive strategies including role play, periods of guided reflection and discussions. The educational workshop intervention was part of a multifaceted approach to implementing self-directed ACP workbooks called Conversation Starter Kit (CSK) (Kaasalainen, Sussman et al., 2021; Sussman, Kaasalainen et al., 2021). CSK booklets were created for use by either residents or their family care partners, when residents may not possess the capacity to complete the CSK booklet activities. CSK booklets extend a previously tested evidence-based palliative program known as, Strengthening a Palliative Approach in Long-Term Care (SPA-LTC) (Kaasalainen et al., 2020). This paper includes findings related to the following research questions: What is the impact of an educational workshop on LTC staff’s (a) level of knowledge related to a palliative approach to care and (b) perceptions regarding having ACP conversations?

## Methods

### Study Design and Participants

A single group, pre-test and post-test design was used to evaluate the change in scores in LTC staff’s knowledge about implementing a palliative approach to care and perception toward ACP conversations as a result of the educational workshop. All study procedures were approved by the Office of Research Ethics Board at McMaster University. Informed consent was obtained from all study participants. This study was conducted within three LTC home sites in two cities in Southern Ontario, where the educational workshop was delivered as part of implementing the SPA-LTC program within each LTC home including the CSK booklet (Kaasalainen, Sussman et al., 2021). The workshop was conducted once in each of the three LTC homes, and all categories of staff were invited to attend in-person using posters and emails from the administrators of the homes. Those who attended received a certificate of completion. Staff members attended based on their availability on the day of the training. Participants included multidisciplinary staff that included nurses (Registered Nurses (RNs), Registered Practical Nurses (RPNs), personal support workers (PSWs) and other registered staff (i.e., social workers, physiotherapists, dietitians), and support staff (i.e., dietary aides, housekeeping, laundry, recreational therapists, maintenance, office/administration).

### The Intervention: Educational Workshop on Palliative Approach to Care and ACP Communication

The educational workshop was designed to complement the implementation of the CSK booklets in order to prepare LTC staff understand the concept of palliative approach to care, common stigma and misunderstandings. The workshop was a necessary first step prior to handing the CSK booklets to residents and/or family care partners by the research team. The workshop served to provide staff with basic knowledge and skills to effectively engage with residents and family if they had questions, concerns or reflections as a result of reviewing the CSK booklet as opposed to feeling unprepared. Essentially, the intervention was comprised of a 1-day educational workshop divided into two sessions (Table 1). The first session consisted of an interactive communication workshop involving a didactic presentation and role-playing activity. The following topics were covered: how to implement a palliative approach to care through ACP, goals of care conversations and EOL planning, common communication challenges that may arise (i.e., family disagreements), how to support others within the LTC homes when a resident dies (i.e., other residents) and the need for staff self-care. The second session included an overview of ACP, including the implementation of the CSK booklet (Kaasalainen, Sussman et al., 2021). In addition to the workshop, a champion team was created with the participating LTC homes, who regularly met with the research team to share progress and challenges of CSK implementation.

**Table 1.** Educational Workshop Overview.

Educational training session	Session 1: Interactive Communication Workshop on implementing a palliative approach to care and EOL planning	Session 2: Overview of ACP and introduction of an ACP tool, the CSK
Lead by	Guest educator	SPA-LTC research team
Length	3 hr	30 min
Components	PowerPoint Videos, and poetry Role playing	PowerPoint slides Role playing
Topics of discussion	- Importance of early conversations with residents and families (goals of care) - Common challenges (i.e., family care partner disagreements in goals of care) - Strategies to reduce common challenges (i.e., practicing empathy) - Role-playing (i.e., conversations with a family care partner on a resident’s declining health status) - Supporting others (i.e., supporting residents when a co-resident dies) - Staff self-care (i.e., the Acknowledge, Debrief, and Dispose process)	- Overview of SPA-LTC project - Overview of ACP - ACP tools including CSK - Role playing activity with opportunity to collaborate as a team on ACP discussions

*Note*. CSK = Conversation Starter Kit; ACP = Advance Care Planning; SPA-LTC = Strengthening a Palliative Approach in Long-Term Care.

### Measures

Sociodemographic data were acquired from all participants. Preliminary efficacy outcome measures were collected within a month prior to the implementation of the educational workshop and a month after the educational workshop intervention. All measures were collected using a hard copy of the data collection form available in the LTC homes, with the help of the champions, while maintaining the confidentiality of all the participants.

### Preliminary Efficacy

Two outcomes were measured to assess the preliminary efficacy of the educational workshop at baseline and 1-month post-intervention. Knowledge regarding implementing a palliative approach to care was assessed using the End-of-Life Professional Caregivers Survey (ELPCS) (Lazenby et al., 2012) and changes in staff perception toward ACP conversations were assessed using the Staff Perceptions Survey (SPS) (Haras, 2013; Haras et al., 2015).

The ELPCS is a 28-item self-reporting survey used to assess the palliative care-specific educational needs of multidisciplinary professionals (Lazenby et al., 2012). In a validation study, the ELPCS demonstrated strong internal consistency (Cronbach’s alpha = 0.96) (Lazenby et al., 2012). The ELPCS has been widely used and tested in a variety of patients and cultural contexts (Block et al., 2016; Kaasalainen et al., 2017; O’Shea et al., 2017; Wallace et al., 2018). It consists of three subscales: Patient-and Family-centered communication (i.e., encouraging patients/families to complete ACP) as depicted in the item, “I am comfortable talking to patients and families about personal choice and self-determination”; Cultural and Ethical Values (i.e., being present with dying patients), for example, “I am comfortable dealing with patients’ and families’ religious and cultural perspectives”; and Effective Care Delivery (i.e., personal resources), as depicted in the following item, “I feel that my workplace provides resources to support staff who care for dying patients” (Lazenby et al., 2012). Respondents are asked to rate each item on a 5-point Likert scale ranging from 1 suggesting the lowest level of skill to 5, suggesting the greatest level of skill (Lazenby et al., 2012). This measure was used in the present study prior to and post-intervention to gage participants’ knowledge regarding a palliative approach to care. Given that the higher scores indicates higher knowledge regarding a palliative approach to care, it is hypothesized that scores will increase from baseline to post educational workshop intervention.

The SPS is a 30-item measure used to explore staff’s Knowledge, Attitude, Comfort, and Support as factors to assess perceptions about ACP in nephrology nurses (Haras, 2013; Haras et al., 2015). Therefore, the SPS was used to explore LTC staff’s perceptions of ACP in this study prior to and post-intervention. This measure was originally validated in a sample of nephrology nurses and showed strong internal consistency (Cronbach’s alpha = 0.92) and high subscale reliability ranging between 0.84 and 0.97 (Haras, 2013; Haras et al., 2015). Each item is scored on a scale from 1 to 4 to demonstrate how much they agree or disagree with each statement (Haras, 2013; Haras et al., 2015). For example, “ACP helps direct medical care of the patient”; and “I see myself as patient advocate by initiating ACP discussions.” As such, higher scores post-intervention would indicate LTC staff’s increased perceptions of readiness, and agreements with ACP conversations as increase in knowledge of ACP and access to support have been identified as important in contributing toward overall perception about ACP (Haras et al., 2015).

### Statistical Analysis

Data was analyzed using the SPSS IBM Statistical Software version 28.0 with an α value of <0.05. Results are presented as percentages, mean, and standard deviation (SD). Paired sample *t*-tests were completed to compare the mean score on the two measures before and after the educational workshop and changes in outcome measures (difference, 95% confidence interval). Cohen’s *d* was used to calculate the preliminary effect sizes of changes.

## Results

### Participant Characteristics

Participant characteristics are summarized in Table 2. A total of 40 LTC staff completed the study. The most common profession was nurses (40%) and the staff were predominantly female (82.5%). The average age of LTC staff was 43.2 years old (±10.8 years). The total average number of years worked in LTC among staff was 14 years (±11.9 years). Most participants were employed full-time (90%). On average, 51.3% had previously completed formal ACP training.

**Table 2.** Participant Characteristics.

Participant characteristics	(*N* = 40)
	Mean (SD)
Age (in years)^a^	43.21 (10.80)
Years worked in LTC	14.05 (11.86)
Years worked in this LTC home	8.19 (6.51)
Sex	*n* (%)
Male	7 (17.5)
Female	33 (82.5)
Ethnicity^a^	n (%)
Aboriginal	0 (0)
Black	3 (7.6)
White	15 (38.5)
Asian-Chinese	8 (20.5)
South Asian	11(28.2)
West Indian	1 (2.5)
Hispanic	1 (2.5)
Religion^a^	*n* (%)
Roman Catholic	16 (40)
Protestant Christian	7 (17.5)
Muslim	2 (5)
Jewish	1 (2.5)
Buddhist	1 (2.5)
No religious affiliation	6 (15)
Seventh-Day Adventist	3 (7.5)
Orthodox	2 (5)
Christian	1 (2.5)
Employment Status	*n* (%)
Part-Time	4 (10)
Full-Time	36 (90)
Occupation	*n* (%)
NUR	
Registered Nurse	6 (15.0)
Registered Practical Nurse	10 (25.0)
PSW	
PSW	4 (10)
REG	
Social Worker/Social Service Worker	1 (2.5)
Physiotherapist	1 (2.5)
Physiotherapist Assistant	1 (2.5)
Dietitians	2 (5.0)
SS	
Dietary Aide	1 (2.5)
Recreational therapist	9 (22.5)
Office/Administration	5 (12.5)
“Have you had formal ACP Training?”^a^	*n* (%)
No	19 (48.7)
Yes	20 (51.3)

*Note*. NUR = nurses; PSW = personal support worker; REG = registered staff; SS = support staff.

a Missing for one participant.

### Preliminary Efficacy

Post-intervention scores on the ELPCS indicate that across all LTC staff categories there was an increase in ELPCS scores suggesting improved knowledge in implementing a palliative approach to care (p ≤ .0001, t = −5.01). There was also a significant difference in the mean scores on the SPS suggesting staff’s increased perceptions of readiness, opinions, and agreements with ACP statements post-intervention (p ≤ .027, t = −2.297) (See Table 3 for Change in Outcomes post-educational workshop intervention).

**Table 3.** Change in Outcomes Post-Educational Workshop Intervention.

Survey	LTC Staff (*n* = 40)			Significance	Effect sizes
	Baseline Mean (SD)	Post-Intervention Mean (SD)	Change (95% CI)		
ELPCS^a^	2.27 (0.91)	2.78 (0.75)	−0.51 (0.64)	*p* < .001	−0.799 (−1.152, −0.439)
SPS^b^	3.00 (0.39)	3.12 (0.29)	−0.12 (0.34)	*p* = .027	−0.363 (−0.681, −0.041)

a End-of-Life Professional Caregiver Survey; Higher scores on this measure indicate respondent’s self-reported *increase in* knowledge regarding implementation of *a* palliative approach to care.

b Staff Perceptions Survey; Higher scores on this measure indicate higher perceptions of readiness, opinions, and agreements with ACP statements.

## Discussion

The educational workshop intervention in this study illustrated statistically significant improvements in LTC staff’s knowledge and comfort in implementing a palliative approach to care and their perceptions toward ACP conversations. Currently, in Canada, the LTC system is not structured effectively to have these ACP conversations early in the disease trajectory (Siu et al., 2020; Wong et al., 2021). Earlier ACP can potentially mitigate future family conflicts or poor care decisions (Andreasen et al., 2019). Ideally, being prepared in advance can assist LTC staff in being guided by prior ACP discussions when the resident is no longer of the capacity to make decisions or if family care partners feel uncomfortable in making these decisions, instead of guessing solely on personal experiences (Andreasen et al., 2019). Preparing in advance can strengthen the trust in relationships between residents, family care partners, and LTC staff, and improve the overall experience of living and dying in LTC (Andreasen et al., 2019). ACP discussions can serve as a precursor for a seamless transition to EOL care informed by residents’ previously expressed values and wishes (Pritchett et al., 2021). Yet, ACP discussions remain uncommon in LTC leading to stress, guilt, poor decision-making confidence in family care partners and inappropriate transfers to the hospital including admission to critical care in the terminal phase of life (Siu et al., 2020; Wong et al., 2021).

It is imperative that multidisciplinary staff within LTC are provided ongoing access to education regarding ACP. Formal education provided within LTC directly for multidisciplinary staff is more effective in comparison to staff seeking education outside the work environment (e.g., online modules, independent learning, etc.) as this can ensure a collective quality of understanding of ACP concepts (Cloutier et al., 2021; Gilissen et al., 2018; Kaasalainen et al., 2017). Having multidisciplinary LTC staff receive the same level of education can promote comfort, knowledge, facilitation of best practices and collaboration in developing competencies related to a palliative approach to care within their unique work environment (Anstey et al., 2016; Frey et al., 2020; Iida et al., 2021; Pereira et al., 2021; Sarakbi et al., 2022). The findings of this study are comparable to other studies involving educational workshops that included multi-modal (e.g., didactic lecture, interactive role-playing) approaches being more advantageous for learners when compared to one mode of delivery because they can be suitable for different learning styles (Forsetlund et al., 2021; Shorey et al., 2021).

Due to the impact of the COVID-19 pandemic, there is a high turnover rate of staff in LTC, thus it is important to offer educational workshops on a regular basis to ensure the continuity of ACP and EOL communication competencies (Hunter et al., 2020; Kaasalainen et al., 2017; White et al., 2021). LTC staff have experienced unprecedented levels of stress, disenfranchised grief and compassion fatigue due to an exponential increase in resident death, staff shortage and unmanageable workload (Hunter et al., 2020; White et al., 2021). Hence, incorporating the component of self-care with palliative education where they can also learn about resources and support strategies (i.e., grief support) can be beneficial for LTC staff (Hunter et al., 2020). With self-care strategies, LTC staff can learn to maintain personal well-being during their professional practice and reduce the likelihood of burnout (Hunter et al., 2020).

As evidenced by the study results, preparing LTC staff with a formal educational workshop may improve comfort with ACP involving residents and their family care partners; therefore, lessening the avoidance of this topic altogether and instead enhancing engagement (Hunter et al., 2020; Jeong et al., 2011; Kaasalainen et al., 2017; Lam et al., 2018; Lin et al., 2019; Spacey et al., 2021; Sussman et al., 2017; Vellani, Green et al., 2022). Despite the benefits of having early ACP, these conversations continue to not be consistently addressed between LTC staff, residents and family care partners (Berning et al., 2021; Sussman et al., 2020). Therefore, formal educational workshops can serve as a facilitator for ACP (Lam et al., 2018). Facilitating earlier ACP can benefit LTC staff, residents, and family care partners in integrating a person-centered palliative approach to care (Zhou et al., 2022). Timely ACP discussions can also promote the seamless transition to EOL ensuring goals of care are achieved as desired by the residents (Aasmul et al., 2018; Howard et al., 2021; Pritchett et al., 2021).

This educational workshop provided an opportunity in valuing each multidisciplinary LTC staff’s role by combining their expertise and acknowledging their inclusivity when participating in future ACP and EOL communication (Vellani, Green et al., 2022). Therefore, this study could inform further research on evaluating educational workshops that promotes multidisciplinary collaboration in implementing a palliative approach to care while also catering to individual learning needs.

### Limitations

One limitation of this study is its small sample size affecting the generalizability of the results. The participants may not be representative of the LTC staff population; for instance, no external consultants, agency or casual staff participated in this study. No staff identified as belonging from Indigenous communities. Future work should make dedicated efforts on using sampling approaches that allow for a more diverse sampling representative of the LTC workforce. There were also fewer PSWs than other categories of staff in this study. PSWs provide direct care for LTC residents and are sought out for their knowledge regarding residents among family care partners (Vellani, Puts et al., 2022). Therefore, there is a critical need for educational initiatives involving PSWs as an important members of the multidisciplinary team to impact the implementation of a palliative approach to care. Future projects should also examine the impact of a staff category and extent of direct involvement in resident care, gender and ethnicity on their scores. As well, complement quantitative data with qualitative data to acquire staff’s perspectives that may not get captured in selected measures. Data should also be collected to identify changes in scores on knowledge and perception in relation to the larger SPA-LTC program implementation that includes a variety of components as previously identified as well as creation of the champions team in the home. Finally, workshop sessions were often scheduled at times that not all staff were able to attend, which may have impacted turnout. Hence, the SPA-LTC team has now created online webinars that incorporate interactive components, which staff can access at their own time.

## Conclusions and Implications

Overall, the educational workshop was effective in improving knowledge about a palliative approach to care and perceptions related to ACP communication among LTC staff. Future evaluation of ACP and structured educational workshops are recommended in LTC homes across Canada and elsewhere, which should also include examining the impact on practice of ACP discussions initiated by staff and its impact on residents, care partners and health systems outcomes. Formal educational workshops such as the one assessed in this study have the potential to promote continuity in implementing a palliative approach to care in LTC and promote end-of-life care that is informed by residents’ expressed wishes and values.

There is a need for LTC homes in Canada and elsewhere to initiate ACP discussions early when residents are able to express their values and wishes as a critical component of implementing a palliative approach to care (Hunter et al., 2020; Sussman et al., 2020; Sussman, Kaasalainen et al., 2021). There is a compelling need for encouraging and recommending future practices within policy work to include ACP communication and to normalize and standardize such discussions as part of routine practice (Zhou et al., 2022). Our study has promising results and adds to the discourse related to the role of educational endeavors to increase multidisciplinary staff’s comfort with ACP discussions in LTC.
